# The Role of Breastfeeding on Respiratory Outcomes Later in Childhood

**DOI:** 10.3389/fped.2022.829414

**Published:** 2022-04-28

**Authors:** Paola Di Filippo, Mauro Lizzi, Massimiliano Raso, Sabrina Di Pillo, Francesco Chiarelli, Marina Attanasi

**Affiliations:** Department of Pediatrics, University of Chieti, Chieti, Italy

**Keywords:** breastfeeding, human milk, prematurity, DLCO, lung function, breast milk, children, FeNO

## Abstract

**Background:**

Breastfeeding is associated with a lower risk of wheezing in early childhood, but its effect later in childhood remains unclear. We investigated the association of breastfeeding and respiratory outcomes in children aged 11 years.

**Materials and Methods:**

We performed an observational longitudinal study including 110 prepubertal children. Information about breastfeeding duration, wheezing and asthma was collected by questionnaires. At 11 years of age, we measured spirometry parameters, lung volumes, diffusing lung capacity, and fractional exhaled nitric oxide. We used logistic and linear regression models to examine the associations of breastfeeding duration with the odds of asthma and lung function measures. All multivariable analyses were adjusted for sex, smoking during pregnancy, gestational age at birth, twins, and mode of delivery (confounder model).

**Results:**

Breastfeeding duration was associated with FEV_1_ z-score [β = 0.04, CI 95% (0.02–0.09)], FEF75 z-score [β = 0.06, CI 95% (0.03–0.09)] and FEV_1_/FVC z-score [β = 0.03, CI 95% (0.00–0.07)], but not with diffusing lung capacity and fractional exhaled nitric oxide. No association of breastfeeding duration with preschool wheezing, ever asthma and current asthma was documented.

**Conclusion:**

We showed that children breastfed for longer time presented higher FEV_1_, FEV_1_/FVC, and FEF75 z-score values at 11 years of age compared to children breastfed for shorter time, suggesting a protective effect of breastfeeding on airways, and not on lung parenchyma (lung volumes and alveolar capillary membrane) or allergic airway inflammation. The positive effect of breastfeeding duration on lung function lays the foundation to promote breastfeeding more and more as effective preventive measure.

## Introduction

In literature, the benefits of breastfeeding on children psychophysical development, nutrition and immune system have been widely demonstrated (1, 2). Therefore, the World Health Organization and the American Academy of Pediatrics recommend exclusive breastfeeding for the first 6 months of life and partial breastfeeding for the first year and beyond (3).

Currently, the impact of breastfeeding on respiratory health is less clear. The putative association between breastfeeding and lung function could be explained by epigenetic effects and the modulation of gut microbiota, lung growth and immune system (4).

It is generally recognized that breastfed infants have less frequent and less severe respiratory infections than non-breastfed infants (5–7). Indeed, human milk provides immunological benefits through a direct protection of specific components (lactoferrin, lysozyme, defensine, and other cytokines), and through the stimulation of the immune system due to its high content of growth factors and nucleotides (8).

Recently, it was supposed that breastfeeding might also have a direct effect on lung growth (2). Ogbuanu et al. (9) stated that breastfeeding effect on respiratory system might be the result of complex interactions between the protective immunoactive factors and the mechanical effect. The latter consists of a more protracted suckling at the breast compared with the bottle, which could determine an increased lung capacity in breastfed compared with bottle-fed children.

Lower breastfeeding rates were documented in preterm infants compared to children born at term, and gestational age at birth was considered a strong predictor of breastfeeding initiation (10, 11). In Italy, the Italian Health Institute report 11/44, including 3,235 preterm newborns from 56 Neonatal Intensive Care Units, confirmed that exclusively breastfeeding depended on the gestational age (12). In addition, in our previous study (13) we showed lower lung diffusing capacity (DLCO) z-score levels in ex-preterm children compared to children born at term later in childhood. Therefore, the relationship between breastfeeding and respiratory outcomes is more difficult to investigate in preterm infants than healthy controls.

To date, studies showing the effect of breastfeeding on lung function have reported contrasting results. Most of them found higher forced vital capacity (FVC) or forced expiratory volume in 1 s (FEV_1_) in previously breastfed school-aged children (9, 14–17).

On the other hand, Guilbert et al. (14) reported a decreased FEV_1_/FVC ratio in 1,246 breastfed infants, particularly for those children born from asthmatic mothers, suggesting a negative effect of breastfeeding in this subgroup.

However, no studies have examined the association of breastfeeding duration with DLCO later in childhood. In addition, several studies were limited by several methodological issues, such as different confounding or modifying factors considered and heterogeneity of the study populations (9, 14).

Recently, Miliku and Azad (4) tried to explain the significant heterogeneity regarding breastfeeding and asthma development between studies. Firstly, the authors stated that it was impossible to perform a randomized control trial due to unethical reason. Secondly, the heterogeneity in asthma and breastfeeding assessment, in study settings, in breastfeeding culture, and human milk composition were also potential source of bias across the different observational research studies. Lastly, Kusunoki et al. mentioned the possibility of reverse causation that occurs when the outcome precedes and causes a change in the exposure, as in premature wheezing leading to breastfeeding prolongation (18).

The primary aim of this study was to evaluate the effect of breastfeeding duration on respiratory outcomes later in childhood. As secondary outcome we also examined the mediating role of breastfeeding duration in the association between gestational age at birth and lung function, specifically diffusing capacity of the lungs which was lower in ex-preterm children compared to controls, as described in our previous study (13).

## Materials and Methods

### Study Design and Population

**FIGURE 1:**
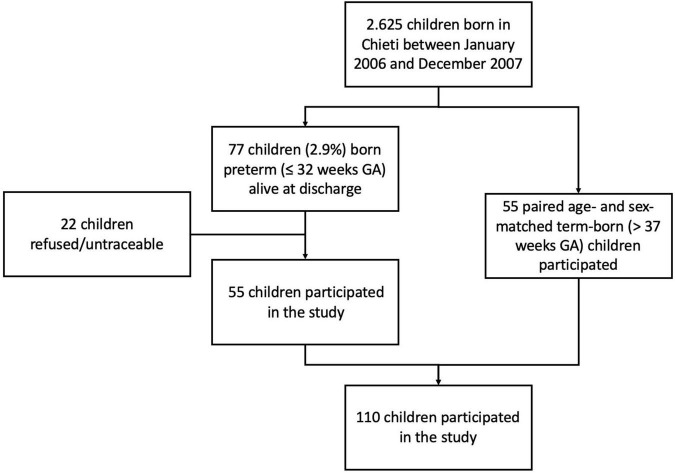
Flow chart of the study. GA, gestational age.

The study was carried out at the Pediatric Allergy and Respiratory Unit of the University of Chieti. The original study was not designed specifically for analyzing the association between breastfeeding and respiratory outcomes. All characteristics of the study population were described previously in our study (13). In Table 1 we summarized those mother and child characteristics which were interesting for this study aim. In particular, we carried-out an observational longitudinal study including 110 prepubertal children, of whom 55 were ex-preterm children born ≤ 32 weeks of gestational age and 55 had no past history of prematurity, followed from birth to childhood (Figure 1). Asthma and atopy were not exclusion criteria. The study was approved by the Ethical Committee of University of Chieti (protocol number 4,205) and written consent was obtained from the parents of the enrolled children.

**TABLE 1:** Characteristics of children and their mothers.

	All	Term-born children	Ex-preterm children	*p*
Subjects	110	55	55	
**Child’s characteristics**				
Sex (%)				1.000
Male	54 (49.1)	27 (49.1)	27 (49.1)	
Female	56 (50.9)	28 (50.9)	28 (50.9)	
Gestational age at birth (weeks)	34.4 (25.6 − 41.3)	38 (36 − 41.3)	31.1 (25.6 − 32.9)	**<0.001^§^**
Birthweight (Kg)	1.8 (0.9 − 3.9)	3.2 (1.6 − 4.1)	1.4 (0.7 − 2.2)	**< 0.001^§^**
Anthropometric data at 11 years of age				
Weight (Kg) Height (cm) BMI (kg/m2)	42.8 ± 9.8 146.7 ± 9.5 19.6 ± 3.2	43.3 ± 9.7 148.2 ± 10.7 19.6 ± 3.1	42.3 ± 10.0 145.3 ± 8.1 19.8 ± 3.4	0.640 0.490 0.710
Twins (%)				**<0.001**
Yes	19 (17.3)	0 (0)	19 (34.6)	
No	91 (82.7)	55 (100)	36 (65.4)	
Breastfeeding (months)	4.5 (0.0 − 24)	12 (0.0 − 24)	2 (0.0 − 24)	**<0.001^§^**
Passive smoking (%)				0.324
Yes	41 (37.3)	18 (32.7)	23 (41.8)	
No	69 (62.7)	37 (67.3)	32 (58.2)	
Pet keeping (%)				0.124
Yes	48 (43.6)	20 (36.4)	28 (50.9)	
No	62 (56.4)	35 (63.6)	27 (49.1)	
SPT (%)				0.550
Positive	39 (35.45)	18 (32.7)	21 (38.2)	
Negative	71 (64.55)	37 (67.3)	34 (61.8)	
Bronchiolitis (%)				0.541
Yes	12 (10.9)	7 (12.7)	5 (9.1)	
No	98 (89.1)	48 (87.3)	50 (90.9)	
Pneumonia (%)				0.751
Yes	11 (10.0)	6 (10.9)	5 (9.1)	
No	99 (90.0)	49 (89.1)	50 (90.9)	
Preschool wheezing (%)				**0.006**
Yes	19 (17.3)	4 (7.3)	15 (27.3)	
No	91 (82.7)	51 (92.7)	40 (72.7)	
Ever asthma (%)				0.547
Yes	10 (9.1)	4 (7.3)	6 (10.9)	
No	100 (90.9)	51 (92.7)	49 (89.1)	
Current asthma (%)				0.696
Yes	7 (6.4)	4 (7.3)	3 (5.4)	
No	103 (93.6)	51 (92.7)	52 (94.6)	
**Maternal characteristics**				
Age (years)	31.1 (5.0)	30.8 (5.4)	31.3 (4.8)	0.593^#^
Caesarean section (%)				**<0.001**
Yes	72 (65.5)	18 (32.7)	54 (98.2)	
No	38 (34.5)	37 (67.3)	1 (1.8)	
Smoking during pregnancy (%)				0.541
Yes	12 (10.9)	5 (9.1)	7 (12.7)	
No	98 (89.1)	50 (90.9)	48 (87.3)	
Maternal asthma (%)				0.449
Yes	19 (17.3)	11 (20.0)	8 (14.5)	
No	91 (82.7)	44 (80.0)	47 (85.4)	
Comorbidities during pregnancy* (%)				**<0.001**
Yes	57 (51.8)	16 (29.1)	41 (74.6)	
No	53 (48.2)	39 (70.9)	14 (25.4)	
Family history of asthma (%)				0.815
Yes	23 (20.9)	12 (21.8)	11 (20.0)	
No	87 (79.1)	43 (78.2)	44 (80.0)	
Family history of inhalant allergy (%)				0.733
Yes	37 (33.6)	18 (32.7)	19 (34.6)	
No	73 (66.4)	37 (67.3)	36 (65.4)	
**Lung function and airway inflammation measures**				
FEV_1_ (Z-score)	0.6 (1.1)	0.7 (0.9)	0.5 (1.3)	0.359^#^
FVC (Z-score)	0.2 (0.9)	0.2 (0.6)	0.2 (1.2)	0.784^#^
FEV_1_/FVC (Z-score)	0.6 (1.0)	0.7 (0.9)	0.6 (1.0)	0.692^#^
FEF_75_ (Z-score)	1.2 (1.0)	1.3 (0.8)	1.2 (1.1)	0.414^#^
FEF_25–75_ (Z-score)	0.4 (0.8)	0.4 (0.6)	0.3 (0.9)	0.207^#^
TLC (%)	98 (71 − 142)	102 (71 − 142)	94 (79 − 141)	**0.016**
RV (%)	101.5 (43 − 248)	111 (29 − 159)	99 (43 − 326)	0.078
sRaw (%)	175 (110 − 354)	173 (110 − 205)	181.5 (109 − 355)	0.056
DLCO (Z-score)	–0.2 (1.3)	0.4 (1.1)	–0.8 (1.2)	**<0.001^#^**
FeNo (ppb)	9.3 (3.8 − 27.2)	10 (5.4–20)	9.1 (3.8–27.2)	0.453**^§^**

Data are presented as n, mean ± SD, n (%) or median (1–99% range). SPT: skin prick test; * comorbidities in pregnancy included gestational diabetes, risk of miscarriage, premature rupture of membranes, gestosis. FEV_1_, forced expiratory volume in 1 s; FVC, forced vital capacity; FEF_75_, forced expiratory flow at 75% of FVC; FEF_25–75_, forced expiratory flow at 25–75% of FVC; TLC, total lung capacity; RV, residual volume; sRaw, specific airway resistance; DLCO, diffusing capacity for carbon monoxide; FeNO, fractional exhaled nitric oxide; ppb, parts per billion. Bold formatting to values where p-value is < 0.05. p-values from Pearson’s Chi-squared test. ^#^p-values from Unpaired t-test. ^§^ p-values from Main-Whitney U-test.

### Breastfeeding, Respiratory Health Outcomes, and Covariates

At the follow-up visit (median age 11 years; 1–99% range 10–12.5 years) an accurate family and personal medical history was collected by a pediatric pulmonologist. Questionnaires at 11 years of age provided information about child’s breastfeeding by asking to the primary caregiver (most commonly mothers) about the age of the infant when last given breast milk, without distinguishing between exclusive and non-exclusive breastfeeding up to what age the infant was breastfed. Any breastfeeding was considered as a continuous variable in months.

Information on maternal age, smoking during pregnancy, passive smoking, pet keeping, time of weaning, child’s ethnicity, and family history of asthma and allergy was obtained by parental questionnaires. Information on mode of delivery, child’s sex, gestational age and birthweight was extracted from medical records.

Information on preschool wheezing, ever physician-diagnosed asthma and current asthma were obtained at age 11 years by questionnaires. We defined preschool wheezing as physician-diagnosed wheezing from birth to 5 years of age. We defined current asthma as ever diagnosis of asthma with either wheezing or medication use in the past 12 months. All questions on wheezing and asthma were based on the International Study on Asthma and Allergy in Childhood (ISAAC) questionnaire (19). Anthropometric parameters (height, weight, BMI) and pubertal stage were assessed by a clinical evaluation at the research center. Allergic sensitization was assessed by skin prick test for the most common inhalant allergens (grass, house dust mite, cat, and dog dander, mugwort, ragweed, molds). Histamine (10 mg/ml) and saline were considered as positive and negative controls respectively; diameters ≥ 3 mm were considered positive (20).

### Lung Function and Airway Inflammation Outcomes

At the visit, participants were in stable clinical condition without having experienced any respiratory disease in the previous 2 weeks.

We assessed lung function by flow/volume curves according to ATS/ERS guidelines (21). The main spirometric parameters included were: FEV_1_, FVC, FEV_1_/FVC ratio, forced expiratory flows between 25 and 75% of the FVC (FEF_75_, FEF_25–75_). Standardized body plethysmography was used to measure Total Lung Capacity (TLC), specific airways resistances (sRaw) and residual volume (RV). Each patient performed lung function measurement at least 3 times; the maximal tolerated variability for the 3 lung function evaluations was considered less than 10% (22).

Diffusing lung capacity test (DLCO) was measured with a standardized single breath technique (Vmax^®^ Autobox V62J, Carefusion, Hoechberg, Germany) according to ERS/ATS recommendations (23). Nobody had a history of anemia.

We used prediction equations from the Global Lung Initiative (GLI-2012) (24, 25) and specialized software (26) to calculate Z-scores for DLCO, FEV_1_, FVC, FEF_75_, FEF_25–75_, and FEV_1_/FVC. The lower limit of normal (LLN) was considered at the 5th percentile of the z-score distribution (24) which corresponded to –1.64. TLC, RV, sRaw were expressed as percentages of predicted for age, height, sex and ethnicity according to GLI-2012 reference values (25).

We assessed fractional exhaled nitric oxide (FeNO) with an on-line method using a single breath exhalation and a sensitive chemiluminescence assay (Ecomedics CLD 88) according to ATS-ERS recommendations (27).

### Statistical Analysis

Continuous data were presented as mean and standard deviation or median and range 5–95%. Categorical data were presented as numbers and percentages. We created age- and sex-adjusted z-scores for BMI according to the Italian reference data (28). In addition, we compared the characteristics of ex-preterm children and those born at term by using independent samples *t*-tests, Mann-Whitney *U*-tests and Pearson’s Chi-squared tests.

Spearman correlation was performed to investigate the relationship among different covariates (gestational age at birth, birthweight, bronchopulmonary dysplasia (BPD), duration of mechanical ventilation).

We used logistic and linear regression models to examine the association of breastfeeding duration with the odds of asthma and lung function measures, respectively. All multivariable analyses were adjusted for sex, smoking during pregnancy, gestational age at birth, twins and mode of delivery (confounder model). Confounders were selected firstly from literature (29–31) and subsequently tested for their association with both the determinant and the outcome, or a change of the unadjusted effect estimates of ≥10% when added to the univariate model. All measures of association are presented as odds ratios or z-scores and their corresponding 95% confidence intervals.

We examined if the association between gestational age at birth and DLCO, already shown in our previous study (13), was explained by breastfeeding duration; we performed mediation analysis using PROCESS macro v4.0 for SPSS (32).

The statistical significance level was *p* < 0.05. SPSS version 25.0 for Windows (IBM, Armonk, NY, United States) and STATA/IC 15.1 (StataCorp, 2017. *Stata Statistical Software: Release 15*. StataCorp LLC. College Station, TX, United States) were used to perform statistical analyses.

## Results

### Subject Characteristics

All participants were Caucasian and pre-pubertal at the follow-up visit.

Among participants 10/110 (9.09%) were never breastfed, 34/110 (30.9%) were breastfed for the first 3 months of life, 19/110 (17.27%) stopped breastfeeding between 3 and 6 months of life, 47/110 (42.73%) were breastfed for more than 6 months. Children with a previous history of prematurity were breastfed for less time compared to children born at term (median breastfeeding duration 2 months, 1–99% range (0.0–17) vs. 12 months, 1–99% range (0.5–24); *p* < 0.001, respectively). Importantly, there were no differences between two groups for mother history of asthma, family history of asthma and allergy, smoking during pregnancy, passive smoking, pet keeping, and for lower respiratory tract infections (pneumonia and bronchiolitis). At follow-up visit, there were no differences for BMI z-score, allergy sensitization by skin prick test, eosinophil blood count, airway inflammation, and lung function parameters, except for DLCO z-score between ex-preterm children and those born at term [–0.80 (0.16) vs. 0.44 (0.15), respectively; *p*-value < 0.001]. In addition, there was no difference for ever asthma and current asthma, except for preschool wheezing between ex-preterm children and those born at term (27.3% vs. 7.3%, respectively; *p*-value = 0.006).

### Breastfeeding Duration and Respiratory Outcomes

In the crude model we found that there was an association of breastfeeding duration with DLCO z- score [β = 0,06, CI95% (0.02–0.09)], FEV_1_ z-score [β = 0.04, CI95% (0.01–0.07)], FEF75 z-score [β = 0.03, CI95% (0.00–0.06)], sRaw% [β = –1.27, CI95% (–2.39 to –0.16)], but there was no association with FVC z-score [β = 0.02, CI95% (–0.01–0.05)], FEV_1_/FVC z-score [β = 0.02, CI95% (–0.01–0.05)], FEF25-75 z-score [β = 0.01, CI 95%(–0.02–0.03)], TLC% [β = 0.44, CI 95% (0.00–0.88)], VR% [β = 0.77, CI 95%(–0.56–2.09)], and FeNO [β = 0.12, CI 95% (–0.05–0.28)]. In the confounder model, after adjusting for sex, gestational age at birth, twins, smoking during pregnancy, and mode of delivery, we found that the previous associations persisted statistically significant for FEV_1_ z-score [β = 0.04, CI 95% (0.02–0.09)], FEF75 z-score [β = 0.06, CI 95% (0.03–0.09)] and for FEV_1_/FVC z-score, albeit weakly [β = 0.03, CI 95% (0.00–0.07)]. The data was shown in Table 2.

**TABLE 2:** Association of breastfeeding duration with respiratory parameters.

		FEV1 Z-score (95% CI)	FVC Z-score (95% CI)	FEV1/FVC Z-score (95% CI)	FEF_75_ Z-score (95% CI)	FEF_25–75_ Z-score (95% CI)	TLC% (95% CI)	VR% (95% CI)	sRaw% (95% CI)	DLCO Z-score (95% CI)	FeNO (ppb) (95% CI)
**Subjects**		110	110	110	110	110	108	108	108	108	106
CRUDE MODEL *p-value*	110	0.04 (0.01 − 0.07) ***p* = *0.016***	0.02 (–0.01–0.05) *p* = *0.155*	0.02 (–0.01 − 0.05) *p* = *0.197*	0,03 (0.00 − 0.06) ***p* = *0.027***	0,01 (–0.02 − 0.03) *p* = *0.617*	0,44 (0.00 − 0.88) *p* = *0.050*	0,77 (–0.56 − 2.09) *p* = *0.255*	–1,27 (–2.39 − –0.16) ***p* = *0.026***	0,06 (0.02 − 0.09) ***p* = *0.001***	0,12 (–0.05 − 0.28) *p* = *0.176*
CONFOUNDER MODEL *p-value*	110	0.04 (0.02 − 0.09) ***p* = *0.003***	0.03 (–0.00–0.06) *p* = *0.071*	0.03 (0.00 − 0.07) ***p* = *0.049***	0.06 (0.03 − 0.09) ***P* < *0.001***	0.02 (–0.00 − 0.05) *p* = *0.085*	0.45 (–0.07 − 0.97) *p* = *0.090*	0.48 (–1.09 − 2.05) *p* = *0.546*	–0.91 (–2.21 − 0.39) *p* = *0.166*	0,05 (–0.05 − 0.07) *p* = *0.078*	0.14 (–0.52–0.33) *p* = *0.152*

*Data are presented as z-score derived from linear regression model; FEV_1_, forced expiratory volume in 1 s; FVC, forced vital capacity; PEF, peak expiratory flow; FEF_75_, forced expiratory flow at 75% of FVC; FEF_25–75_, forced expiratory flow at 25–75% of FVC; TLC, total lung capacity; RV, residual volume; sRaw, specific airway resistance; DLCO, diffusing capacity for carbon monoxide; FeNO, fractional exhaled nitric oxide. Confounder model is adjusted for sex, smoking during pregnancy, gestational age, twins and mode of delivery. Bold formatting to values where p-value is <0.05.*

We decided to not include in the model as confounders birthweight, bronchopulmonary dysplasia, mechanical ventilation duration and pregnancy complications to avoid the multicollinearity. Spearman correlation showed a strong correlation of gestational age at birth with birthweight (rho = 0.88; *p*-value < 0.001), BPD (rho = –0.84; *p*-value < 0.001), mechanical ventilation duration (rho = –0.67; *p*-value < 0.001) and pregnancy complications (rho = –0.44; *p*-value < 0.001).

No association of breastfeeding duration with preschool wheezing, ever asthma and current asthma was found both in the crude model and after adjusting for confounders. The data was shown in Table 3.

**TABLE 3:** Association of breastfeeding duration with preschool wheezing and asthma.

		Preschool wheezing OR (95% CI)	Ever asthma OR (95% CI)	Current asthma OR (95% CI)
Subjects		110	110	110
**Crude model** *p*-value	110	0.98 (0.91 − 1.06) *p* = *0.658*	0.96 (0.86 − 1.07) *p* = *0.414*	0,96 (0.85 − 1.09) *p* = *0.525*
**Confounder model** *p*-value	110	1.20 (0.99 − 1.22) *p* = *0.091*	*0.95* (*0.83–1.07*) *p* = *0.363*	0.92 (0.78–1.09) *p* = *0.332*

*Data are presented as odds ratio derived from logistic regression model; Confounder model is adjusted for sex, smoking during pregnancy, gestational age, twins, and mode of delivery. p values are expressed in italic.*

### Mediation Analysis

**FIGURE 2:**
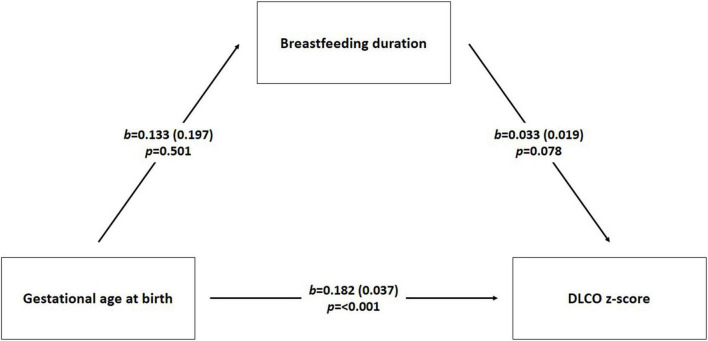
Mediation analysis scheme. We performed mediation analysis using linear regression to investigate the interrelationship of three numeric variables DLCO z score (diffusing lung capacity) as dependent variable, breastfeeding duration (months) as mediator, and gestational age at birth (weeks) as independent variable. The model was also adjusted for gender, smoking during pregnancy, twins, and mode of delivery. b is the regression coefficient; *p*-value was considered significant < 0.05.

After finding that in confounder model breastfeeding duration was associated with FEV_1_ z-score, FEV_1_/FVC z-score and FEF_75_% z-score, but not with DLCO z-score, we specifically investigated if the association between gestational age at birth and DLCO z-score, already described previously (13), could be explained by the mediating role of breastfeeding. We illustrated the mediation analysis in the Figure 2.

The path (direct effect) from gestational age at the birth to breastfeeding duration was negative but not significant [β = –0.133, standard error (se) = 0.197; *p*-value = 0.501]. The path (direct effect) from gestational age at birth to DLCO z-score was positive and statistically significant (β = 0.182, se = 0.037; *p*-value < 0.001) indicating that children with higher gestational age at birth could more likely show higher DLCO z-score values compared to children with lower gestational age at birth. The path (direct effect) from breastfeeding duration to DLCO z-score values was positive but not significant (β = 0.033, se = 0.019; *p*-value = 0.078).

The indirect effect is tested using non-parametric bootstrapping. If the null 0 falls between the lower and the upper bound of the 95% confidence interval, then the inference is that the population indirect effect is 0. If 0 falls outside the confidence interval, then the indirect effect is inferred to be non-zero (33). In this case the indirect effect (IE = –0.004) is negative but not significant: 95%CI = (–0.024-0.008).

## Discussion

In our study population we found a dose-dependent association of breastfeeding duration with FEV_1_ z-score, FEF_75_ z-score, and for FEV_1_/FVC z-score at 11 years of age, but no association with DLCO z-score values, preschool wheezing, asthma and airway eosinophilic inflammation. Importantly, we showed that those children breastfed for longer time presented higher FEV_1_, FEV_1_/FVC, and FEF75 z-score values compared to children breastfed for shorter time.

Few studies evaluated the association between breastfeeding and lung function parameters later in childhood. In 54,000 children aged 8–12 years randomly selected from the ISAAC Phase II, breastfeeding was associated with higher predicted FEV_1_ (15). Tennant et al. (17) showed that breastfeeding duration less than 4 weeks was a predictor of lower FEV_1_ at 14 years of age in 252 members of the Newcastle Thousand Families Study cohort. Similarly, lung function evaluation in 1,033 children aged 10 years reported that FEV_1_ was increased by 39.5 ml in children breastfed for at least 4 months compared with those not breastfed (9). The protective effects of breastfeeding on lung function may be due to reduced respiratory infections (34) and greater height in breastfed children (35). In addition, the benefit of a longer duration of breastfeeding on lung capacity measured at 10 years of age persisted at 18 years of age (16). In contrast with our findings, these authors showed that breastfeeding duration was associated with lung volumes rather than FEV_1_, suggesting a positive effect of breastfeeding on lung parenchyma and not on respiratory airways (16).

On the other hand, several studies found opposite results. Breastfeeding duration was not associated with lung function outcomes in a prospective cohort study with 377 healthy children with low risk for asthma at 6 years of age (29). At the same way, no association of breastfeeding with respiratory outcomes was found in 620 children with a family history of allergic disease at 12 and 18 years of age (36). These contrasting findings were probably due to heterogeneity in sample size, definition of determinant, age of participants, confounders, and study methodology.

Long-chain polyunsaturated fatty acids (LCPUFAs) are essential for structural and functional integrity of the endothelial system (37); phytochemicals, such as polyphenols, flavonoids, and carotenoids, have antioxidant and vascular health promotion activity (38); angiopoietins are important for endothelial cell survival and proliferation, periendothelial cell recruitment, and vascular stability (39). We hypothesized that aforementioned substances could have a benefit on the development of the capillary alveolar membrane. For this reason, we investigated the potential effect of breastfeeding duration on DLCO z-score values.

In our study population, we speculated that the crude association of breastfeeding duration with DLCO z-score values was influenced by the presence of ex-preterm children who were breastfed for less time than full-term infants. In addition, mothers of premature infants produce breast milk characterized by a different composition, as demonstrated by Yesildal et al. (39): they found lower ANG-1 levels in human milk of 9 mothers with preterm delivery (≤33 weeks) compared to 17 mothers with term and late preterm delivery (>33 weeks).

However, positive association of breastfeeding duration with DLCO z-score values resulted not statistically significant after adjusting for confounders (sex, gestational age at birth, twins, smoking during pregnancy, and mode of delivery). Gestational age at birth persisted as predictor affecting diffusing lung capacity independently from other covariates. To better study the association among breastfeeding, gestational age at birth and DLCO z-score values, we also examined if the association of gestational age at birth with DLCO z-score values was explained by breastfeeding duration. We confirmed a positive direct effect of gestational age at birth on DLCO z-score values without a mediating role of breastfeeding duration. Therefore, diffusing lung capacity was influenced by prematurity (13), not by breastfeeding duration.

In literature, few studies investigated the association of breastfeeding with FeNO, a marker of eosinophilic airway inflammation. Den Dekker et al. (40) showed that no breastfed children had lower FeNO levels compared to breastfed children; the authors supposed that a shorter breastfeeding duration was associated to lower occurrence of respiratory tract infections leading to a neutrophilic airways inflammation. At the same way, in a cohort-prospective study, Gorlanova et al. (29) found no effect of breastfeeding duration on FeNO levels in 377 healthy term infants at 6 years of age. According to these findings, we showed no association of breastfeeding duration with FeNO. In addition, we also observed no association of breastfeeding with SPT results. Based on these findings, we could hypothesize a greater effect of breastfeeding on airways development compared to the effect on allergic airway inflammation.

Several studies showed that breastfeeding could be a protective factor for wheezing in the first years of life.

Dell and To (41) found that a shorter breastfeeding duration was a risk factor for wheeze in the first 2 years of life in 331,100 Canadian children. Kull et al. (42) reported that exclusive breastfeeding for at least 4 months was a protective factor for asthma development at the age of 4 years in a birth cohort of 4,089 children. Oddy et al. (43) showed that the introduction of milk in association to breast milk before 4 months of age was a risk factor for asthma and atopy at 6 years of age in a prospective cohort study including 2,187 Australian children. More recently, Den Dekker et al. (40) reported that not breastfed participants had increased risk of late (wheezing > 3–6 years) and persistent wheezing (wheezing ≤ 3 years and > 3–6 years) and that a shorter breastfeeding duration was associated with current asthma at age 6 years in a Dutch prospective cohort study with 5,675 children.

Generally, it is recognized that breastfeeding protects against wheezing in early childhood (9, 44–46), mostly when it is associated to lower respiratory tract infections. These advantages in breastfed children seem to be mostly mediated by an immunomodulating effect of breast milk (47). Indeed, breast milk contains immunoglobulins, lactoferrin, oligosaccharides, and maternally derived leukocytes and cytokines. These bioactive substances influence the naïve cells of the immature infant immune system, especially in its early development. Growth factors, cytokines, and miRNAs contained in human milk could stimulate leukocyte differentiation inducing viral clearance, tissue repair or regulation of disease pathway and immunological memory (35, 47). Reducing respiratory tract infections, these immune mechanisms could have a prognostic effect on later lung function (35, 48).

A non-immune effect of breast milk was also mentioned in literature. For example, colostrum is rich in growth factors like TGF- β, and the amount of these molecules gradually decreases in the milk over the first months of life (49). These growth factors could lead to a beneficial lung development increasing the elastin activity in fibroblasts (35, 50). In addition, the mechanical effect of suckling during breastfeeding contribute to respiratory muscles training (35, 51) and increased lung capacity (9).

Although these putative theories, the precise mechanisms through which lung function is improved by breastfeeding remain unclear.

The role of breastfeeding as a preventative strategy for asthma in later childhood is less clear.

Two systematic reviews synthesized the current evidence on this topic. Dogaru et al. (31) analyzed 117 studies and showed that the protective effect of breastfeeding on asthma was more pronounced during the first 2 years of life, although still evident at school age. Lodge et al. (52) found in 29 studies that a longer breastfeeding duration was associated with a reduced risk of asthma between 5 and 18 years of age.

On the contrary, the Mater-University of Queensland Study of Pregnancy on 4,964 children showed no association between the breastfeeding duration and the prevalence of asthma at 14 years of age (53).

The protective effect of breastfeeding on asthma mostly in early childhood and not in later life, could be due to a decreasing effect of breastfeeding over time because of other superadded factors, such as atopy passive smoking, viral respiratory illnesses and obesity (31, 54). Aforementioned data suggest that the primary protective effect of breast milk is mostly on viral wheezing rather than atopic asthma (29, 46). Dogaru et al. (31) hypothesized that the protective effect of breastfeeding in infants continued in older children, given that lower respiratory tract infections in early life influenced the development of asthma in later childhood (34).

In contrast to our association of breastfeeding duration with lung function, we found no association of breastfeeding with wheezing and asthma. First of all, this suggests that the better lung function of previously breastfed infants compared to non-breastfed ones may not be clinically relevant at 11 years of age; it would be interesting to investigate whether this can be highlighted in later ages. Then, sample size and recall bias might also explain the absence of this association. In addition, we defined preschool wheezing as referred to the first 5 years of life without distinguishing into wheezing phenotypes. For this reason, we didn’t know how many children wheezed during the first 2 years of life when the stronger effect of breastfeeding on wheeze was mostly evident (31, 46, 55).

The major strength of this study is the use of detailed respiratory outcomes (spirometry parameters, lung volumes, DLCO, FeNO), the adjustment for relevant confounders. To the best of our knowledge, this is the first study that evaluated the effect of breastfeeding on lung diffusing capacity. In addition, the use of z-score for spirometric and DLCO parameters minimizes different effects of age, sex, height, and race.

Some limitations need to be discussed. First of all, sample size was small and the study was performed in a population that also included ex-preterm children reducing generalizability to the general population. Second, information on breastfeeding duration, wheezing and asthma was obtained by questionnaires, which might have led to reporting recall bias. For wheezing and asthma, however, validated and widely accepted ISAAC questionnaire was used. Third, although several potential confounders were taken into account, residual confounding might be a distortion as in any observational study. Last, the absence of a lung function evaluation at birth. However, we compared lung function parameters in ex-preterm children with those at term at 11 years of age.

## Conclusion and Future Perspectives

We found that breastfeeding is associated in dose-dependent manner with lung function parameters later in childhood. Importantly, we showed that those children breastfed for longer time presented higher FEV_1_, FEV_1_/FVC, and FEF75 z-score values at 11 years of age compared to children breastfed for shorter time. In addition, we observe a protective effect of breastfeeding on airways, and not on lung parenchyma (lung volumes and alveolar capillary membrane) or allergic airway inflammation.

The positive effect of breastfeeding duration on lung function lays the foundation to promote breastfeeding more and more as effective preventive measure. Our findings confirm that breastfeeding is an early-life exposure that may influence the developmental programming of respiratory outcomes.

Therefore, in all infants breastfeeding should be encouraged to prevent the risk of lower lung function later in life.

However, several methodological issues and biological variability in human milk limit the generalizability of our results. Further studies are needed to better investigate the protective effects of breastfeeding on lung function and parenchyma, and allergic airway inflammation.

## Data Availability Statement

The raw data supporting the conclusions of this article will be made available by the authors, without undue reservation.

## Ethics Statement

The studies involving human participants were reviewed and approved by the Ethics Committee of the University of Chieti. Written informed consent to participate in this study was provided by the participants’ legal guardian/next of kin.

## Author Contributions

PD: data collection, database creation, and writing – original draft preparation. ML: tables creation. MR: tables creation. SD: revision of the manuscript. FC: revision of the manuscript. MA: statistical analysis, writing, and revision. All authors contributed to the article and approved the submitted version.

## Conflict of Interest

The authors declare that the research was conducted in the absence of any commercial or financial relationships that could be construed as a potential conflict of interest.

## Publisher’s Note

All claims expressed in this article are solely those of the authors and do not necessarily represent those of their affiliated organizations, or those of the publisher, the editors and the reviewers. Any product that may be evaluated in this article, or claim that may be made by its manufacturer, is not guaranteed or endorsed by the publisher.
